# 1224. The incidence of severe *C. striatum* hospital-acquired pneumonia sharply increased and was associated with a high mortality rate

**DOI:** 10.1093/ofid/ofac492.1056

**Published:** 2022-12-15

**Authors:** Yun Woo Lee, Jin Won Huh, Sang-Bum Hong, Jiwon Jung, Min Jae Kim, Yong Pil Chong, Sung-Han Kim, Heungsup Sung, Kyung-Hyun Do, Sang-Oh Lee, Chae-Man Lim, Yang Soo Kim, Younsuck Koh, Sang-Ho Choi

**Affiliations:** Asan Medical Center, Seoul, Seoul-t'ukpyolsi, Republic of Korea; Asan Medical Center, Seoul, Seoul-t'ukpyolsi, Republic of Korea; Asan Medical Center, Seoul, Seoul-t'ukpyolsi, Republic of Korea; Asan Medical Center, Seoul, Seoul-t'ukpyolsi, Republic of Korea; Asan Medical Center, Seoul, Seoul-t'ukpyolsi, Republic of Korea; Asan Medical Center, Seoul, Seoul-t'ukpyolsi, Republic of Korea; Asan medical center, Seoul, Seoul-t'ukpyolsi, Republic of Korea; Asan Medical Center, Seoul, Seoul-t'ukpyolsi, Republic of Korea; Asan Medical Center, Seoul, Seoul-t'ukpyolsi, Republic of Korea; Asan Medical Center, Seoul, Seoul-t'ukpyolsi, Republic of Korea; Asan Medical Center, Seoul, Seoul-t'ukpyolsi, Republic of Korea; Asan Medical Center, Seoul, Seoul-t'ukpyolsi, Republic of Korea; Asan Medical Center, Seoul, Seoul-t'ukpyolsi, Republic of Korea; Asan Medical Center, Seoul, Seoul-t'ukpyolsi, Republic of Korea

## Abstract

**Background:**

The clinical information on patients with severe *C. striatum* pneumonia who require intensive care unit admission is currently limited.

**Methods:**

We investigated the incidence and characteristics of severe *Corynebacterium striatum* pneumonia during a 6-year period at Asan Medical Center in comparison with severe pneumonia associated with MRSA.

**Results:**

Between 2014 and 2019, there were 27 adult cases of severe *C. striatum* pneumonia. The majority of the cases (70.4%) were hospital-acquired pneumonia (HAP), and about half of the patients (51.9%) were immunocompromised. The incidence of *C. striatum* HAP significantly increased from 1.0% (2/200) in 2014-2015 to 5.4% (10/185) in 2018-2019 (*P* < 0.001), while the incidence of severe methicillin-resistant *Staphylococcus aureus* (MRSA) HAP significantly decreased from 12.0% to 2.7% during the same period. Of the 75 HAP cases whose bacterial pathogens were identified in 2018–2019, *C. striatum* was responsible for 13.3% of the cases. The 90-day mortality rates were similarly high in the *C. striatum* and MRSA groups (59.3% vs. 50.5%, *P* = 0.42).

**Figure:**
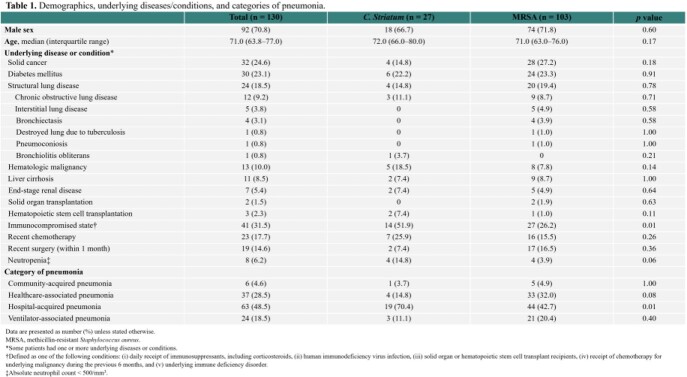

**Figure:**
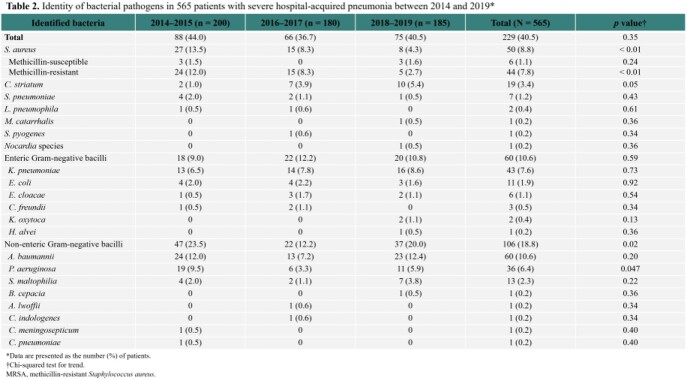

**Figure:**
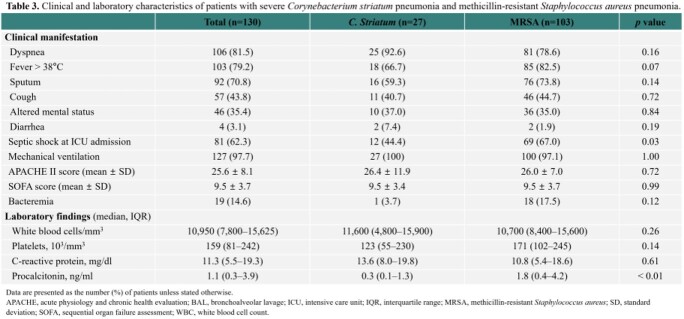

**Conclusion:**

In conclusion, *C. striatum* was a major pathogen of recent severe HAP and was associated with a substantially high mortality rate.

**Disclosures:**

**All Authors**: No reported disclosures.

